# Validity of self-reported weight, height and resultant body mass index in Chinese adolescents and factors associated with errors in self-reports

**DOI:** 10.1186/1471-2458-10-190

**Published:** 2010-04-12

**Authors:** Xiaoyan Zhou, Michael J Dibley, Yue Cheng, Xue Ouyang, Hong Yan

**Affiliations:** 1 From the Department of Epidemiology and Health Statistics, School of Public Health, Xi'an Jiaotong University College of Medicine, No.76 West Yanta Road, Xi'an, China; 2 Sydney School of Public Health, University of Sydney, Room 307A, Edward Ford Building (A27), University of Sydney, Sydney, NSW 2006, Australia

## Abstract

**Background:**

Validity of self-reported height and weight has not been adequately evaluated in diverse adolescent populations. In fact there are no reported validity studies conducted in Asian children and adolescents. This study aims to examine the accuracy of self-reported weight, height, and resultant BMI values in Chinese adolescents, and of the adolescents' subsequent classification into overweight categories.

**Methods:**

Weight and height were self-reported and measured in 1761 adolescents aged 12-16 years in a cross-sectional survey in Xi'an city, China. BMI was calculated from both reported values and measured values. Bland-Altman plots with 95% limits of agreement, Pearson's correlation and Kappa statistics were calculated to assess the agreement.

**Results:**

The 95% limits of agreement were -11.16 and 6.46 kg for weight, -4.73 and 7.45 cm for height, and -4.93 and 2.47 kg/m^2 ^for BMI. Pearson correlation between measured and self-reported values was 0.912 for weight, 0.935 for height and 0.809 for BMI. Weighted Kappa was 0.859 for weight, 0.906 for height and 0.754 for BMI. Sensitivity for detecting overweight (includes obese) in adolescents was 56.1%, and specificity was 98.6%. Subjects' area of residence, age and BMI were significant factors associated with the errors in self-reporting weight, height and relative BMI.

**Conclusions:**

Reported weight and height does not have an acceptable agreement with measured data. Therefore, we do not recommend the application of self-reported weight and height to screen for overweight adolescents in China. Alternatively, self-reported data could be considered for use, with caution, in surveillance systems and epidemiology studies.

## Background

The prevalence of overweight and obesity is increasing among children and adolescents worldwide [1-3]. Adolescent obesity in particular has increased rapidly in recent years [4]. Obesity early in life is a risk factor for the development of many chronic diseases such as type 2 diabetes mellitus and cardiovascular diseases [5], and is likely to track into adult life [6]. The large population of China combined with the emerging adolescent obesity epidemic means there is an urgent need for appropriately validated methods to monitor the trends in overweight and obesity in youth, and to evaluate the related health programs. Body mass index (BMI) is most commonly used to define overweight and obesity. Some studies used self-reported weight and height to derive BMI values and estimate the prevalence of overweight and obesity [7-11]. Reported data on weight and height are easier to collect than measured data, and would potentially be suitable for large scale surveillance systems and epidemiological studies. But the inaccuracy of these reported values may skew obesity evaluation, risk factor identification and the evaluation of interventions. Thus, validation studies of reported height and weight are of interest in assessing obesity and overweight in Chinese adolescents.

Previous validation studies that examine the accuracy of self-reported height and weight have applied several different methods. Most validity studies compare mean difference of reported and measured values [12-15]; but correlation coefficients have also been applied widely with some studies using it as the main method [16,17]. Sensitivity and specificity [14,18-20] or chi-square [21] for screening overweight, intraclass correlation coefficient [16,22] and eta-square [21] have also been applied. Bland-Altman's plots of limits of agreement [15,21,23-25] and kappa statistics [13,17,23] have also been used to evaluate validity. However, when the aim was to compare two methods (continuous data); comparison of means tells us little about the accuracy of the methods, and correlation is only a measure of association. The Bland-Altman's plot of limit of agreement is a better method [26], and when screening for overweight, sensitivity and specificity can also be applied.

Previous validation studies have shown inconsistent outcomes in adolescents. Some studies suggested that adolescents' self-reports of height and weight were valid [16,17,19], while others raised concern about the accuracy of self-reported anthropometric values in adolescence [12,14,18,20,21,27-29]. Furthermore, the validity of self-reported height and weight has not been adequately examined in diverse youth samples, especially in different cultural contexts [30]. Most existing studies on adolescents have been conducted in Western populations, while there are no reported validity studies conducted in Asian children and adolescents. Asian body sizes, diet, health practices, and socio-cultural norms are different from those of their Western counterparts. Where studies have been conducted in Asian populations, results diverge from those conducted in the West. For example, previous validation studies in Japanese adults [23] and Japanese adult women [31] have shown more accurate self-reported height and weight than in Western studies. The issue of whether Asian adolescents report their height and body weight with greater accuracy remains under researched

Several previous studies have raised algorithm for correction of self-reported BMI. Most used simple equations[16,32,33], but there is also published research where quadratic equation was used [34]. Age appeared most frequently in the correction equations, while smoking, education, physical activity, self-rated health, body image and ethnicity were also used to adjust reported anthropometric data [16,22,32,33,35]. These previous correction equations were satisfactory and led to more accurate estimations of the mean BMI and obesity prevalence compared to estimates that were calculated directly from reported values [22,32]. However, differences still remained between corrected obesity prevalence and true obesity prevalence. This previous study suggests that equations should not be used across populations [33].

This study aims to examine the accuracy of self-reported weight, height, and resultant BMI values in Chinese adolescents and of the adolescents' classification as overweight or obese. As part of this analysis, we identify the demographic and socioeconomic factors associated with errors in self-reported data.

## Methods

### Subjects and design

This study was performed as a part of a health survey conducted in Xi'an, China. Xi'an is a large city located in northwest China with a population of about seven million. This survey assessed the level of overweight and obesity among adolescents aged 12 to 16, and identified associated environmental and behavioral factors influencing these levels.

The study participants were recruited using a multistage cluster sampling procedure. In the first stage, thirty schools in the city area were randomly selected proportional to the size of their enrollments. One class was then randomly selected in each grade within these selected schools. In the third stage, twenty students were systematically randomly sampled in each class and invited to take part in the survey. The students were asked to answer self-administered questionnaires on diet patterns, physical activity and sedentary activity. The parents of the subject completed a household questionnaire that gathered information about household socioeconomic status, demographic information, parental characteristics, and family environment. The questionnaire on diet patterns asked subjects to report their height and weight. Following the questionnaire, a physical examination took place to measure the height and weight of subjects. Approval from the Xi'an Municipal Bureau of Education and the headmasters of the selected schools were secured beforehand. Information sheets about the study and separate consent forms for both adolescents and their parents were distributed to students at the first visit to the school. All adolescents and their parents signed an informed consent form. This study was conducted between September 2007 and October 2007, with the approval of the Research Ethics Committee of Xi'an Jiaotong University College of Medicine.

### Anthropometric measurements

Anthropometric measurements were performed in an empty room in each school. Subjects were asked to remove all heavy clothes, remove their shoes and undo hair styles and accessories in a preparation area. Next, trained staff weighed the subjects on a calibrated electronic scale (Tanita HD-305) and recorded the value to the nearest 0.1 kilogram. Standing height was measured using a non-stretchable tape suspended from the wall (214 Road Rod™, U.S.A), the subjects stood erect with their shoulders level, hands at their sides, thighs together and heels comfortably together. The subjects also kept their upper back, buttocks and heels in contact with the wall and their head aligned in the Frankfort Plane during the height measurement. The height values were recorded to the nearest 0.1 centimeter. All anthropometric measurements were taken by a single trained staff member.

Body mass index (BMI) was calculated as weight (in kilograms) divided by the height (in meters) squared, for both measured and self-reported heights and weights. Children were classified as overweight and obese using the International Obesity Taskforce (IOTF) age and gender-specified cut-off points, [36] that are based on average percentiles at the age of 18 for BMI values of 25 kg/m^2 ^for overweight and 30 kg/m^2 ^for obesity. These cut-off points have been widely used in studies with children and adolescents [37].

### Demographic and socioeconomic measurements

The adolescents' gender, age and area of residence were included in the self-administered questionnaire. The household questionnaire gave a list of thirteen household facilities (telephone, video cassette player, CD system, DVD player, air conditioner, refrigerator, computer, gas stove, microwave, bicycle, motorbike, car and television) and subjects were asked whether their family owned these facilities. To assess the household economic status, a wealth index was calculated from the list of household facilities using a principal components method to assign a weight for each asset [38]. Scoring factor of the first principal component is the "weight" assigned to each asset in a linear combination of the assets that constitute the index. The index value of each individual was ranked and divided into three categories (tertiles) - the lower, the middle and the higher household economic status.

### Statistical analysis

Differences were calculated as reported values minus measured values in height, weight and BMI. Differences of reported and measured values in height, weight and BMI were determined by the student's paired t-test. Absolute differences of reported and measured values in height, weight and BMI were also calculated. Pearson's correlation coefficients between reported and measured values were calculated, and in order to assess the agreement between the reported and measured anthropometric values, the 95% limits of agreement (LOA) were calculated following the Bland-Altman method [39]. Differences between the reported and measured values were plotted against the means of the reported and measured values, with the mean difference plus or minus 1.96 times its standard deviation. The limits of agreement (LOA) were considered to show "good" agreement if the difference between paired values was approximately equal to one standard deviation (SD) of the mean of the measured values, "fair" agreement is if the width was two SDs, and "poor" if three SDs. Linear regression lines were fitted to the plotted values to test if there was a significant slope indicating a trend in the differences of methods as the mean of the methods increased.

Measured and reported weight, height and resultant BMI values were ranked and divided into quintiles, and quadratic weighted Kappa statistics and their 95% confidence intervals were derived to assess the agreement. Kappa statistics were also calculated to assess the reported and measured BMI, when BMI was divided into three categories based on IOTF cut-off points -normal weight/underweight, overweight and obesity group. Because underweight is rare (only 11 subjects), we combined it into the normal weight group. Quadratic weighted kappa assigns weights to each disagreement pair, with smaller weights indicating smaller agreement, and it is commonly used for ordinal scales. Unweighted kappa, meanwhile, treats all disagreements equally[40]. Unweighted kappa, therefore, is inappropriate for ordinal scales of the present study[41]. The sensitivity, specificity, positive predictive value, and negative predictive value were used to assess the performance of the reported method in screening for overweight and obese individuals. Sensitivity measures the proportion of actual positives that are correctly identified; specificity measures the proportion of negatives which are correctly identified; positive predictive value is the proportion of subjects with positive test results who are correctly diagnosed; while negative predictive value is the proportion of subjects with negative test results who are correctly diagnosed.

Gender, age, household economic status, area of residence and BMI were included in multivariable linear regression models to detect which factors were associated with the difference between reported and measured weight, height and BMI. EpiData 3.1 (Odense, Denmark) was used for data entry, and the analyses were carried out using SPSS 13.0 for Windows (Chicago, U.S.A).

## Results

A total of 1 761 subjects participated in the survey; the consent rate is 97.8% (1761 of 1800). Twenty two participants failed to report their weight and 23 failed to report their height. After the removal of these 35 subjects who failed to report their height and/or weight, 1 726 subjects were included in the analysis. Table 1 shows the characteristics of the study subjects.

**Table 1:** Characteristics of the subjects

Characteristic	N	%
**Gender**		
Boy	822	47.6
Girl	904	52.4
**Age group (years)**		
11.0-12.9	407	23.6
13.0-13.9	490	28.4
14.0-14.9	557	32.3
15.0-16.9	272	15.7
**Household economic status^a^**		
Highest	583	33.8
Middle	588	34.1
Lowest	555	32.2
**Area of residence**		
Urban	1385	80.2
Suburb	341	19.8
**BMI categories^b^**		
Normal weight	1483	85.9
Overweight/Obesity	243	14.1
Total	1726	100.0

^a ^Calculated from a list of 13 household facilities using principal components method [38], then ranked and divided into tertiles.

^b ^Category based on measured weight and height using IOTF cut-off points [36].

The overall mean difference between self-reported and measured values was -2.35 kilograms for weight, 1.36 centimeters for height and -1.23 kg/m^2 ^for BMI. All these differences were statistically significant as determined by the paired t-test. Cohen's d was used as an effect size to indicate the standardized difference between means of reported and measured values. The effect size is 0.217 for the weight, 0.164 for height and 0.393 for BMI. Results showed adolescents significantly underestimated their weight, while they overestimated their height, and the resultant BMI was thus underestimated. Only 46.3% of the adolescents' self-reported weights had an absolute discrepancy within 2.0 kg of the actual value, and only 26.1% were within 1.0 kg. In addition, 54.2% of the subjects' reported heights had an absolute difference within 2 cm of the actual value, and 30.4% were within 1.0 cm. With regard to reported BMI, 71.0% of the adolescents had a gap within 2.0 kg/m^2 ^of the actual BMI, and 41.4% had a gap within 1.0 kg/m^2^.

**Figure 1:**
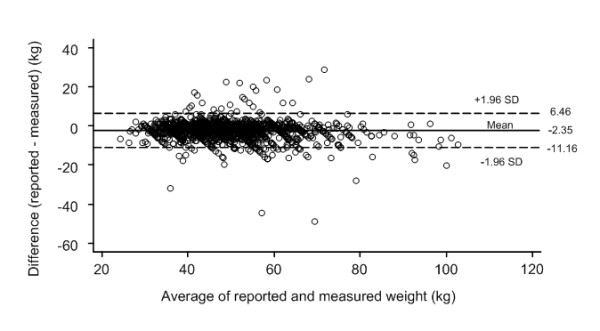
**Bland Altman plot **[39]** of the difference versus the average of reported and measured weights**. Broken lines present 95% limits of agreement, where upper LOA is +1.96 SD and lower LOA is -1.96 SD from mean difference (solid line) of methods.

**Figure 2:**
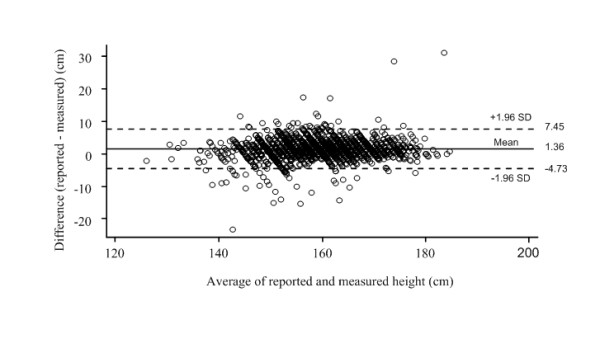
**Bland Altman plot **[39]** of the difference versus the average of reported and measured heights**. Broken lines present 95% limits of agreement, where upper LOA is +1.96 SD and lower LOA is -1.96 SD from mean difference (solid line) of methods.

**Figure 3:**
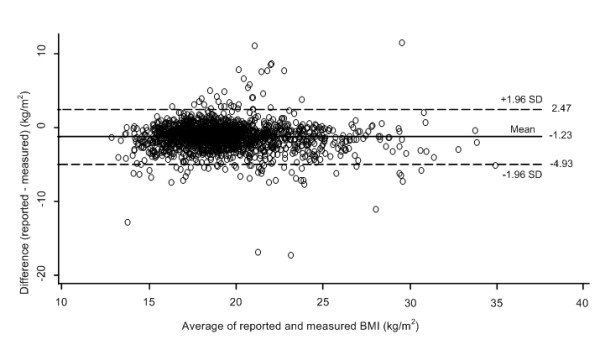
**Bland Altman plot **[39]** of the difference versus the average of reported and measured resultant BMIs**. Broken lines present 95% limits of agreement, where upper LOA is +1.96 SD and lower LOA is -1.96 SD from mean difference (solid line) of methods.

The differences between self-reported and measured values were plotted against the means of the self-reported and measured values for weight (Figure 1), height (Figure 2) and BMI (Figure 3). The 95% limits of agreement were -11.16 and 6.46 for weight, -4.73 and 7.45 for height, and -4.93 and 2.47 for BMI. Thus, 95% of the adolescents' self-reported values fell between 11.16 kilograms below or 6.46 kilograms above the measured value for weight, 4.73 centimeters below or 7.45 centimeters above for height, and 4.93 kg/m^2 ^below or 2.47 kg/m^2 ^above for BMI. The LOA for weight was greater than one SD of the measured weight values (SD 10.3 kg) thus showing only fair agreement; for height the LOA was also greater than one SD of the measured height values (SD 8.7 cm) also showing only 'fair" agreement; but for BMI the LOA was more than two SD of the measured BMI values (SD 3.1 kg/m2) thus showing poor agreement. Thus the LOA for all three anthropometric measurements were sufficiently wide to be regarded as unacceptable, especially BMI.

The slope of the fitted regression line for the values in the BA plot for weight (difference of methods = 0.65-0.06*mean of methods, linear trend p < 0.001) was significant and indicated a slight underreporting at higher weights. There was a similar significant pattern for BMI (difference of methods = -1.31-0.07*mean of methods, linear trend p = 0.006). In contrast the slope of the fitted regression line for height was positive (difference of methods = 1.21+0.11*mean of methods, linear trend p < 0.001) indicating a slight over reporting at higher heights.

The means, mean differences, Pearson correlation coefficients and Kappa statistics for self-reported and measured weight, height and BMI are shown in Table 2. There was no gender effect on the entire above index, thus the results are presented for the total sample. Pearson correlation coefficients between the measured and self-reported values were high for weight and height, but lower for BMI. There were similar patterns for weighted Kappa statistics with the values for height significantly higher than for weight, which in turn was considerably higher than BMI.

**Table 2:** Self-reported and measured weight, height and BMI among adolescents, Xi'an, China

Items	Weight(kg)	Height(cm)	BMI(kg/m^2^)
**Measured value (mean ± S.D.)**	50.5 ± 10.9	158.7 ± 8.4	19.9 ± 3.1
**Reported value (mean ± S.D.)**	48.2 ± 10.3	160.1 ± 8.7	18.7 ± 3.0
**Pearson's correlation^a^**	0.912	0.935	0.809
**Intraclass correlation**	0.85	0.898	0.796
**Weighted Kappa^a, b^**	0.86	0.91	0.75
95% confidence interval	0.84, 0.88	0.90, 0.92	0.74, 0.76
**Mean difference^a, c^**	-2.35	1.36	-1.23
95% confidence interval	-2.56, -2.14	1.21, 1.51	-1.32, -1.14
**Mean absolute difference^a^**	3.36	2.43	1.64
95% confidence interval	3.19, 3.54	2.31, 2.54	1.56, 1.71

^a ^Between reported data and measured data.

^b ^Weight, height and BMI values were ranked and divided into quintiles, and quadratic weighted Kappa statistics are presented.

^c ^Reported data minus measured data.

Using BMI calculated by self-reported height and weight to classify subjects into BMI categories leads to an underestimation in the prevalence of overweight. The prevalence of overweight and obesity from the reported values was 7.5% and 1.5%, respectively; while for the measured values it was 11.4% and 2.5%, respectively. The Kappa value was 0.58 when the three BMI categories from self-reported values were compared to those from measured values. Overall, 8.7% of adolescents were misclassified using their reported values. The screening performance of self-reported and corrected BMI values is presented in Table 3. When overweight and obesity categories were integrated into one category, setting the category from the measured values as the reference, the sensitivity for detecting overweight (including obesity) was low (56.1%), the specificity was high (98.6%), the positive predictive value was low (86.5%) and the negative predictive value was high (93.3%). Correction of the self-reported BMI for age, gender, area of residence and household economic status increased the sensitivity to 82.1%, and the specificity to 97.9%.

**Table 3:** Screening performance of self-reported and corrected BMI value

	measured	reported	Corrected^a^
**Overweight/obese**	243	173	262
**Normal weight**	1483	1553	1464
**Overweight prevalence (%)**	14.1	9.0	15.2
**Sensitivity (%)**	-	56.1	82.1
**Specificity (%)**	-	98.6	97.4
**Positive predictive value (%)**	-	86.5	84.4
**Negative predictive value (%)**	-	93.3	96.2

^a ^Correction of self-reported values for age, gender, area of residence and household economic status.

Multivariable linear regression models including gender, age, area of residence, household economic status, and BMI categories were applied to estimate the effect of these factors. Categorical variables were transferred into dummy variable sets, and these dummy variables were introduced into the regression analysis. Adjusted means (marginal means) were calculated. The data met the assumption of independence, linear and normal distribution of the residuals. The adjusted mean differences between self-reported and measured height, weight and BMI are presented in Table 4. There were no statistically significant differences by gender between the reported and measured weight and height, but there was for BMI. Age had a statistically significant effect for weight, height and BMI. There was an increasing underestimation of self-reported weight and BMI with increasing age, while older adolescents significantly overestimated their height. The area of residence of the adolescents was a significant factor associated with error in self-reporting weight, height and resultant BMI. The subjects living in suburban areas were more likely to underestimate their weight, overestimate their height and report a lower resultant BMI than urban subjects. The difference in self-reported values was higher in overweight/obese adolescents than in adolescents of normal weight, and this was particularly pronounced for boys. Household economic status also affected the difference between measured and reported values in height and resultant BMI, but not in weight. Subjects in families of higher socioeconomic status tended to overestimate their height and underestimate their resultant BMI more than those in families of lower socioeconomic status.

**Table 4:** Adjusted means difference between reported and measured weight, height and BMI

	Weight (kg)		Height (cm)		BMI (kg/m^2^)	
			
Characteristic	Mean (S.E)	P	Mean (S.E)	P	Mean (S.E)	P
**Gender**						
Boy	-2.38 (0.15)	0.479	1.31 (0.11)	0.505	-1.17 (0.06)	0.043
Girl	-2.33 (0.14)		1.41 (0.10)		-1.28 (0.06)	
**Age groups (years)**						
11.0-12.9	-1.94 (0.22)	0.007	1.11 (0.15)	0.029	-1.06 (0.09)	0.009
13.0-13.9	-2.41 (0.20)		1.29 (0.14)		-1.25 (0.08)	
14.0-14.9	-2.35 (0.18)		1.52 (0.13)		-1.25 (0.08)	
15.0-16.9	-2.86 (0.26)		1.52 (0.19)		-1.41 (0.11)	
**Household economic status^a^**						
Poorest	-2.14 (0.18)		1.21 (0.13)		-1.12 (0.08)	
Median	-2.29 (0.18)	0.129	1.38 (0.13)	0.013	-1.23 (0.08)	0.041
Richest	-2.63 (0.18)		1.50 (0.13)		-1.34 (0.08)	
**Area of residence**						
Urban	-2.30 (0.12)	0.015	1.27 (0.08)	0.001	-1.19 (0.05)	<0.001
Suburb	-2.56 (0.24)		1.71 (0.17)		-1.40 (0.10)	
**BMI category^b^**						
Normal	-1.90 (0.11)	<0.001	1.30 (0.08)	0.026	-1.04 (0.05)	<0.001
Overweight/Obese	-5.10 (0.28)		1.74 (0.20)		-2.37 (0.12)	

Mean difference (Mean) and standard error (S.E.) and P value were adjusted by all the other co-variables in multivariable linear regression model.

^a ^Calculated from the list of household facilities using principal components method [38], then ranked and divided into tertiles.

^b ^Category based on measured weight and height using IOTF cut-off points [36].

## Discussion

This study examined the accuracy of self-reported weight, height and resultant BMI in the assessment of the prevalence of overweight in Chinese adolescents. The LOAs between the reported and measured values in this study were unacceptably wide. When reported values were used to classify individuals into BMI categories, the low sensitivity indicated that reported data may not be appropriate to screen for overweight adolescents. The degree of the discrepancy was not affected by gender, but it was associated with area of residence, age and BMI category.

On average, the adolescents' reported weights were underestimated, their heights were overestimated, and their resultant BMI was underestimated. These findings were similar to those in previous studies in adults [24,42-44] and adolescents [16,17,28]. The magnitude of the discrepancies in our study was moderate compared to existing studies in adolescents. In our study, the mean differences were -2.35 kg for weight, 1.36 cm for height and -1.23 kg/m^2 ^for BMI. In comparison, a review of previous studies in adolescents showed a mean difference of -4.0 to 1.5 kg of weight, -1.1 to 6.9 cm of height and -3.0 to 0.2 of BMI value [30].

The Pearson's correlation coefficient between the reported and measured values was high for weight, height and BMI. The Pearson's correlations for weight, height and resultant BMI were consistent with previous studies [16,20,22,28,45]. The weighted Kappa statistics revealed a high level of agreement for weight and height, and substantial level for BMI, a pattern similar to that reported for the correlation coefficients [17]. We also need to mention that quadratic weighted kappa coefficients tend to increase with the number of categories. But, after all, as the number of categories increases, so does the proportion of the variability in the true variable captured by the imperfect ordinal variable[46]. Self-reported data could be considered for use in surveillance systems and large epidemiology studies, given the ease of data collection, its less resource-intensive nature, and high linear correlation and kappa statistics between reported and measured data. But we need to be cautious of the error of reported data, and correlation is a measure of association not agreement. The high correlation could not infer that reported data may be used interchangeably [26].

In the present study, the sensitivity (56.1%) and specificity (98.6%) and positive predictive value (86.5%) in screening for overweight individuals were similar to those in a study of American adolescents [17]. The lower sensitivity was also consistent with previous studies in children from Wales [21], Greece [12] and the Netherlands [14]. These values were lower than those in found studies in three other American adolescent samples [18-20]. Sensitivities in these three studies were about 70%, specificities were above 88%, and positive predictive values were above 80%.

The present study found no significant differences by gender between the self-reported and measured weight and height, however there were differences in BMI. The results for weight and height are consistent with several previous studies, but inconsistent in regards to BMI [12,14,29,47]. However, an earlier study has observed a gender difference in reporting bias for weight and resultant BMI values [19], and another in the correlation between self-reported and measured values for weight and resultant BMI [45]. Age was associated with differences between self-reported and measured values in our study, with older adolescents more likely to exhibit bias than younger ones. This trend is consistent with some previous findings [12,17], although one earlier study found no difference according to age [14], while other research found the same trend for height but the opposite trend for weight [16]. Children categorized as overweight or obese were more likely to underestimate their weight than normal children. This result is consistent with all previous reports [12,14,16,29,47]. Household economic status was not associated with differences between the self-reported and measured values for weight, but it was associated with bias for height and resultant BMI. One previous study found results similar to our findings [16], but another study reported that household economic status was not associated with the difference between self-reported and measured values [19]. We found adolescents living in suburban areas had more bias in their self-reported anthropometric values than those living in urban areas of the city. Previous studies have no information about the effect of area of residence on the report error. It is beyond the scope of the current study to interpret the effects of household economic status and area of residency. Previous studies have indicated that adults [48] and adolescents [49] with a higher socioeconomic status are more concerned about body shape or other peoples' perceptions of their weight. Prior research also shows that rural students are less concerned about weight [50,51]. The difference in concerns about weight may partly explain why household economic status and area of residency were associated with difference between reported and measured values in our study.

Most studies conclude that overweight and/or obese adolescents underreport their body weight and, thus, their resultant BMI, compared to adolescents of normal weight [30]. We had similar findings. We also hypothesized that a population with a relatively thinner body shape or a population with fewer obese people might more accurately report their weight and height. However, the reported errors in our study were moderately to slightly larger than previous studies conducted in Western countries, where the rates of overweight and obesity were higher than in our study. Unexpectedly, we found that adolescents from the suburban areas of the city, a population with a lower prevalence of overweight and obesity [52], reported their body weight and height less accurately compared to their urban counterparts, after adjustment for gender, age, household economic status and BMI status in multivariable linear regression models.

By adjusting the self-reported BMIs for socioeconomic variables, the sensitivity of screening for overweight individuals was increased from 56.1% to 86.6%, and the specificity decreased from 98.6% to 96.4%. Thus, the application of an adjusted formula results in a more accurate identification of overweight adolescents. Nevertheless, the sensitivity does not seem to be sufficient for the identification of overweight individuals even if the reported BMI is adjusted in this way. In addition, the use of the correction formula in this study, or other studies, is limited because the characteristics may differ in different populations or change over time.

One shortcoming of the present study was that our sample was drawn from schools, and adolescents who did not attend a school were not included. The results of the present study will not reflect this relatively small section of the adolescent population. In addition, there was a time interval of about one week between when the students answered the questionnaire and when they were measured. The height and weight of the adolescents may have changed during this week, although this change is likely minimal. Xi'an is a city located in central China. Since China is a vast nation characterized by social, economic, cultural and environmental diversity, the result of this study cannot be generalized to the whole country. However, it may be generalized to several neighbouring big cities that demonstrate similar qualities and patterns.

## Conclusions

Reported weight and height does not have an acceptable agreement with measured data. Therefore we do not recommend the application of self-reported weight and height to screen for overweight adolescents in China. Reported data could be considered for use in surveillance systems and epidemiology studies with caution. Any use of self-reported height and weight data from adolescents in future research studies should be justified with supporting pilot data validating such measures.

## Competing interests

The authors declare that they have no competing interests.

## Authors' contributions

XZ carried out the study, data analyses and drafted the manuscript. MJD participated in study design, data interpretation and helped to draft the manuscript. YC participated in study design, data collection, and made modifications of the paper. OX participated in study design and data collection. HY participated in the study design, data analysis and helped to draft manuscript. All authors read and approved the final manuscript.

## Pre-publication history

The pre-publication history for this paper can be accessed here:

http://www.biomedcentral.com/1471-2458/10/190/prepub
